# Annual transcriptomic trajectory of grapevine buds reveals the coordinated regulation of thiamine and stilbenoid biosynthesis during winter dormancy

**DOI:** 10.1186/s12870-026-08190-w

**Published:** 2026-01-21

**Authors:** Tomas Konecny, Armine Asatryan, Hans Binder

**Affiliations:** 1 Interdisciplinary Centre for Bioinformatics, University of Leipzig, Leipzig, 04107 Germany; 2 Armenian Bioinformatics Institute, Yerevan, 0014 Armenia; 3 Institute of Molecular Biology of National Academy of Sciences, Yerevan, 0014 Republic of Armenia

**Keywords:** *Vitis vinifera*, Bud transcriptomics reanalysis, Machine learning, Self-organizing maps, Thiamine biosynthesis, Stilbenoid biosynthesis

## Abstract

Bud dormancy is a critical survival strategy for perennial plants in temperate climates to endure winter, involving complex transcriptional reprogramming. While previous studies have investigated gene expression during discrete phases of the grapevine bud annual cycle, a holistic understanding requires an integrated, season-wide analysis.

We conducted a meta-analysis by combining and reexamining the transcriptomic data from two seminal studies on grapevine bud development. Using Self-Organizing Maps, we parsed the combined gene expression data into three major clusters corresponding to distinct seasonal activities, which we termed “summer-upregulated,” “winter-upregulated,” and “intermediate.”

Our analysis revealed that genes whose expression was predominantly upregulated during the winter months were significantly enriched in Gene Ontology terms related to stilbenoid biosynthesis and thiamine biosynthesis. This finding prompted a deeper investigation into these two pathways. We observed profound and coordinated upregulation of key genes in both pathways during the coldest winter months, suggesting that these genes are integral components of the bud’s strategy for surviving low temperatures and maintaining viability.

These results confirm the role of stilbenoids as stress-responsive compounds and reveal a novel role for thiamine biosynthesis in the molecular mechanisms of grapevine bud dormancy.

**Supplementary Information:**

The online version contains supplementary material available at 10.1186/s12870-026-08190-w.

## Introduction

Bud dormancy is a key adaptive mechanism in perennial plants that enables survival during adverse seasonal conditions, particularly in temperate climates where cold winters impose physiological constraints on growth. In *Vitis vinifera*, the regulation of dormancy is highly coordinated and aligned with the annual growth cycle, involving both internal and environmental signals that control the timing of bud development, cessation, and reactivation [1–3]. Cold hardiness in grapevine buds is strongly regulated by hormonal signaling, notably the accumulation and signaling activity of abscisic acid, which promotes the acquisition of freezing tolerance and the suppression of premature growth [3]. Dormancy in grapevine buds is classified into three types according to Lang et al. [4]: paradormancy, endodormancy, and ecodormancy, each characterized by a distinct mechanism of growth inhibition and a specific seasonal occurrence. Paradormancy, also known as correlative inhibition, refers to the temporary suppression of bud growth due to physiological signals originating from other parts of the plant rather than from the bud itself. This type of dormancy commonly occurs during spring and early summer, when active shoot growth and apical dominance inhibit the outgrowth of lateral buds. Auxin produced by the apical bud plays a central role in this process, while cytokinins and strigolactones further modulate paradormancy by influencing auxin transport and response [1]. Endodormancy is maintained by internal physiological blocks within the bud that prevent growth even under favorable external conditions. This phase is typically initiated in late summer to early autumn and is triggered by environmental cues such as shortening day length and decreasing temperatures. It is associated with major transcriptional, metabolic, and hormonal changes, which help prepare buds for winter and increase cold tolerance [4, 5]. Ecodormancy, in contrast, is imposed by external environmental conditions, mainly low temperatures, after the internal blocks of endodormancy have been lifted. During late winter to early spring, buds are physiologically capable of resuming growth but remain dormant due to persistent unfavorable conditions. Once temperatures rise and environmental cues become favorable, ecodormancy ends, and bud break occurs [4]. The fulfillment of chilling requirements, often quantified in chilling hours or chill units, is a critical prerequisite for endodormancy release and the transition to ecodormancy. Inadequate chilling under warming climates may lead to earlier or asynchronous bud break, with significant implications for grapevine productivity and regional adaptation [6].

Multiomics studies reveal dormancy as a multilayered regulatory process integrating genomic, epigenetic, transcriptomic, and metabolic networks. Molecular regulators, such as the previously mentioned MADS-box transcription factors and hormone-related genes, govern the developmental transitions associated with grapevine bud dormancy. In parallel, epigenetic mechanisms (including DNA methylation, histone modifications, chromatin remodeling, and small RNAs) modulate chromatin accessibility in response to seasonal cues, providing a form of molecular “memory” that links environmental signals with dormancy status [7–9]. Transcriptomic changes fine-tune the stress response, hormonal signaling, and growth repression [10], whereas metabolic shifts in carbohydrate allocation, antioxidant systems, and secondary metabolite production confer biochemical resilience during winter dormancy [11]. In *Vitis vinifera*, the accumulation of compatible solutes (such as proline, sucrose, and raffinose), soluble sugars, and secondary metabolites enhances cold tolerance in buds, while metabolic adjustment (including sugar metabolism, phenylpropanoid pathways, and stress-response proteins) provides protection against oxidative stress during cold acclimation [12–14]. In addition to classical cryoprotectants, recent studies suggest that vitamins (e.g., thiamine) may act as additional regulators of oxidative balance, suggesting unexplored metabolic dimensions of dormancy [15]. Within this metabolic context, two pathways have emerged as pivotal in grapevine bud resilience: thiamine biosynthesis and stilbenoid metabolism. Thiamine (vitamin B1), notably its active form thiamine diphosphate (TDP), is an essential cofactor in central metabolism and has been shown to function as a signaling molecule that primes plants for both biotic and abiotic stress by enhancing their antioxidant capacity and defense networks [16]. Stilbenoids, such as resveratrol, ε-viniferin, and piceatannol, are phenylpropanoid-derived phytoalexins with potent antioxidant and antimicrobial properties that accumulate in woody tissues and buds during stress and dormancy transitions [9].

In this study, we used an advanced meta-analysis approach using Self-Organizing Maps (SOM) machine learning for reanalysis of transcriptomic datasets (RNA-seq and microarray) from two grapevine studies spanning seasonal bud dormancy transitions [17]. We aimed to identify additional key genes and/or regulatory players that were not the primary focus of the original publications [10, 11]. This approach allowed us to uncover new aspects of metabolic and regulatory coordination, contributing to a deeper understanding of the molecular basis of bud dormancy resistance in grapevines.

## Materials and methods

### Data source

The transcriptomic data used in this meta-analysis were obtained from two publicly available studies (Additional file 1 in [10] and Supplementary Table S3 in [11]). The dataset from Diaz-Riquelme et al. [10]. profiled expression of 23,045 genes in *Vitis vinifera* ‘Tempranillo’ grown in an experimental vineyard at the Instituto Madrileño de Investigación y Desarrollo Rural, Agrario y Alimentario (IMIDRA, Alcalá de Henares, Madrid) and the buds were collected at eight time points over an annual cycle via the Affymetrix Grapegen GeneChip^®^. The dataset from Shangguan et al. [11]. utilized RNA-seq containing 31,858 genes to analyze dormant buds from *Vitis vinifera* ‘Rosario Bianco’ (‘Rosaki’ × ‘Muscat of Alexandria’) grown at the Jiangsu Agricultural Expo Garden in China and collected monthly from November to April at three different nodal positions.

### Data integration

**Fig. 1:**
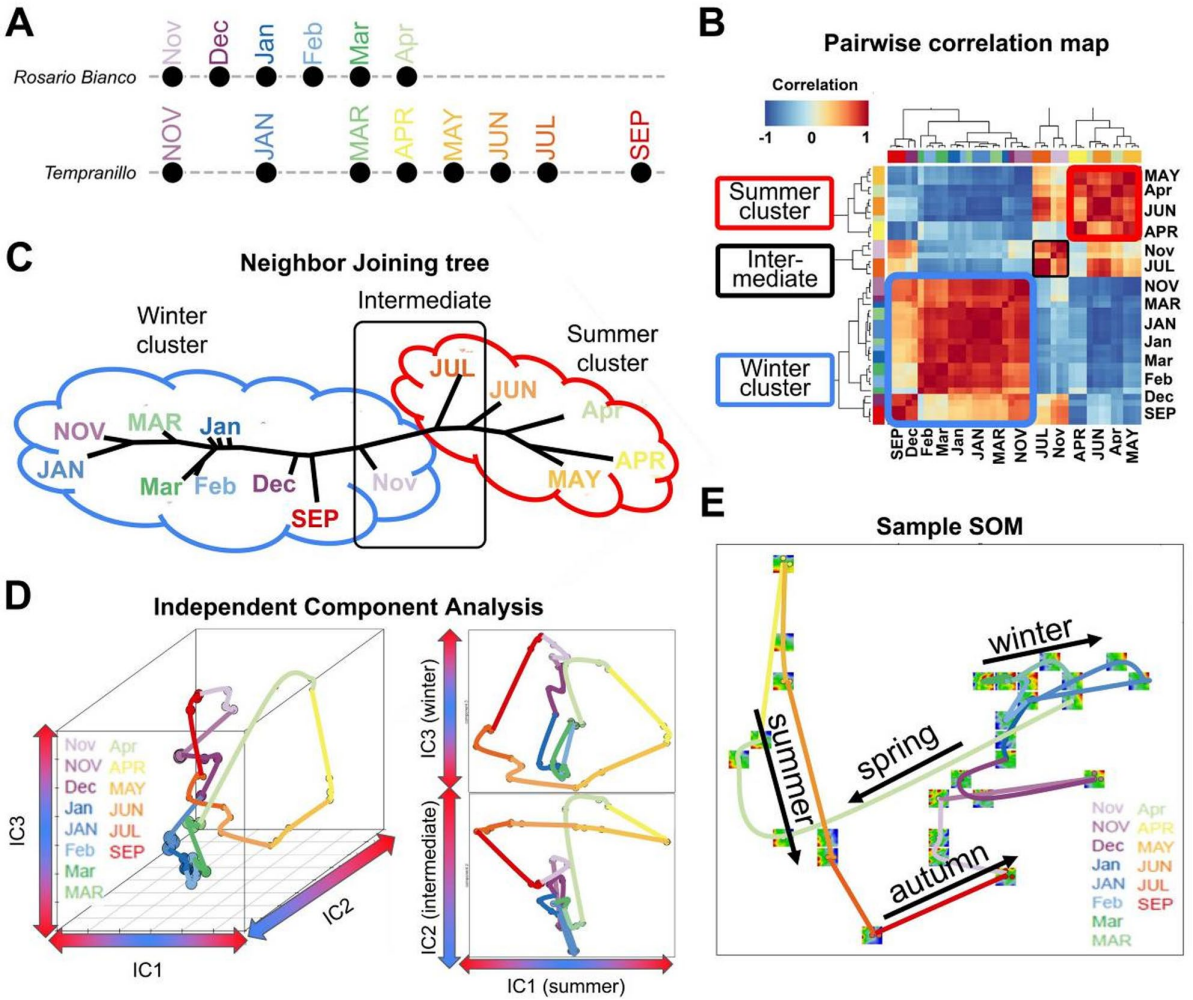
Changing expression patterns across the year in grapevine buds. **A **Overview of the seasonal bud transcriptome sampling timeline in *Vitis vinifera* cultivars ‘Rosario Bianco’ and ‘Tempranillo’, indicating the months during which samples were collected. Samples are displayed according to dataset availability, not strict seasonal sequence. **B** Pairwise correlation map of SOM-weighted eigenvalues of bud transcriptome samples clustered into ‘summer-upregulated’, ‘winter-upregulated’ and ‘intermediate’ transcriptional programs. **C** Neighbor-joining tree of transcriptome samples illustrating the separation of the ‘summer-upregulated’, ‘winter-upregulated’ and ‘intermediate’ clusters. **D** Independent component analysis of bud transcriptomes revealing distinct seasonal trajectories across the IC1, IC2 and IC3 axes. **E** Sample SOM indicating seasonal trajectories in two dimensions

Both bud transcriptome datasets complement each other regarding the chosen months of sampling and, in combination, cover a full annual cycle of bud development (Fig. 1A). Due to the source data limitations, sample collection points in these datasets start in November. Average daily temperatures across sampling points were considered to align datasets for comparative analysis, despite non-standard seasonal ordering (for detailed information about experimental conditions, we refer to the original studies [10, 11]). To facilitate a combined analysis, gene identifiers from both platforms were harmonized on the basis of the *V. vinifera* reference genome annotation (PN40024.v4). Given the different technologies used (microarray, RNA-seq), a normalization strategy had to be applied. Python (version: 3.11.7) implementation of ComBat (pyComBat; version: 0.3.3; [18]) was used to reduce platform-specific biases. The expression values of 16,103 integrated genes were log-scaled after quantile normalization and centralization, ensuring that the subsequent clustering algorithm focused on the overall expression pattern rather than the absolute signal intensity.

### Self-organizing map (SOM) analysis

The integrated and normalized gene expression matrix, containing data points from all expressed genes and all samples in both studies, was subjected to SOM analysis (described in Konecny et al. [19] and [20]).,. This unsupervised clustering technique was used to group genes into 1600 nodes on a two-dimensional quadratic lattice of size 40 × 40, with each node representing a distinct expression profile. Genes with similar expression patterns across the combined time course are mapped to the same or adjacent nodes. This approach allows for the visualization of major transcriptional trends within the dataset. SOM plots, including clustering plots, barplots, pairwise correlation heatmap, neighbor-joining tree (pairwise Pearson correlation distances between SOM cluster centroids), and SOM portraits, were generated by oposSOM package (version 2.2.5) in R (version 4.4.1) [17].

### Gene ontology (GO) and pathway analysis

Following SOM clustering, genes that were mapped to the cluster exhibiting elevated expression during the winter months (November to March) were selected for functional analysis. This “winter-upregulated” cluster was analyzed for GO term enrichment via the PANTHER GO Enrichment tool [21], which applies a false discovery rate (FDR)-corrected p value threshold of < 0.05. On the basis of the significant enrichment of terms related to “thiamine biosynthesis” and “stilbenoid biosynthesis”, the expression profiles of known genes within these pathways were extracted from the normalized dataset and examined in detail to confirm their coregulation during winter dormancy. The genes of the SOM spots were functionally annotated by both the gene set overrepresentation analysis implemented directly in the SOM pipeline and the g: GOSt Functional profiling tool in the g: Profiler [22] with default settings. Pathway-specific genes were obtained from KEGG [23] and VitisNet [24] databases. Custom Python and R scripts were used for visualization of all the plots. The full technical methodology has been described previously [19, 20].

## Results

### Annual trajectory of the bud transcriptome

The pairwise correlation heatmap between the nodes of the SOM portraits of combined data of grapevine bud transcriptomes revealed two major clusters reflecting distinct transcriptional programs (Fig. 1B). One set of correlating samples, virtually spanning May to July is referred to as “summer-upregulated” cluster. The cluster anti-correlates with another set of correlating samples, extending from September to March, referred to as “winter-upregulated” cluster. A third, smaller set of samples consisted of transitional periods between summer-upregulated and winter-upregulated clusters is referred to as “Intermediate cluster” (IMC). A tree-based representation organizes the transcriptomes along a roughly linear backbone, separating the winter-upregulated and summer-upregulated clusters with the IMC in between (Fig. 1C). The independent component (ICA) analysis plot reveals a distinct distribution of the samples along the first three independent components (IC1‒IC3). The distribution results in a slightly more intricate pattern: winter-upregulated transcriptomes are distributed mainly along the IC3 coordinate, whereas summer transcriptomes form an extended loop in the IC1‒IC2 plane, reflecting the activation of distinct transcriptional programs in each season (Fig. 1D). However, this pattern suggests that the transcriptional program transitions in a coordinated and continuous manner across seasons, forming a cyclic expression pattern that mirrors the progression of the annual phenological cycle. Consistent with this, the sample SOM (providing a two-dimensional projection of the multidimensional data space of the samples) projects the annual pattern of the bud transcriptome from spring through summer, autumn, and winter, capturing the full seasonal progression in a two-dimensional plot. The autumn and spring samples occupy intermediate positions, resulting in transition states between the active growth and dormancy phases (Fig. 1E). In summary, the annual dynamics of the bud transcriptome closely follow the seasonal cycle, with clearly distinct transcriptional programs dominating the winter and summer phases.

### Transcriptome trajectory associated with time-dependent expression patterns

**Fig. 2:**
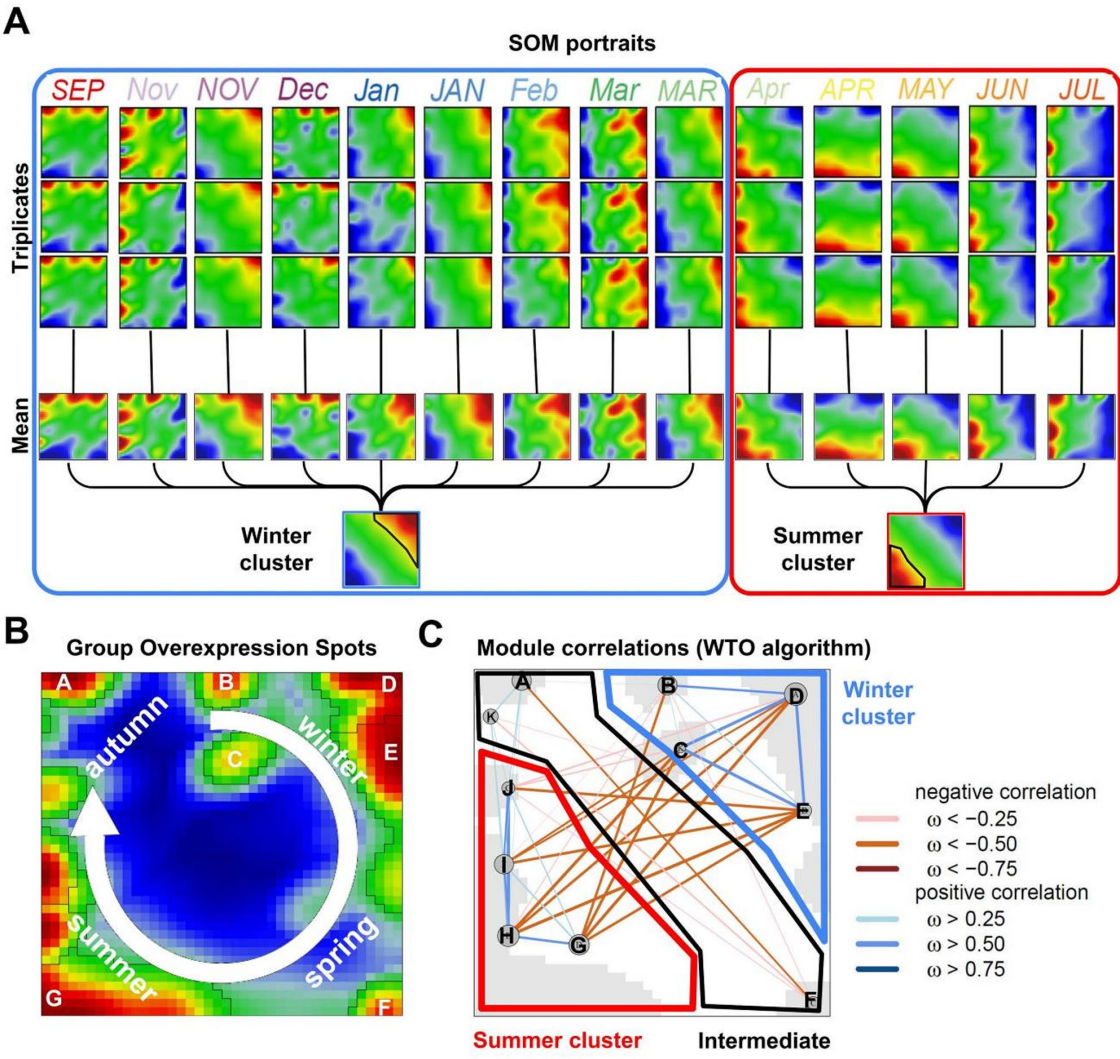
Seasonal transcriptome dynamics reveal annual trajectories. **A** Individual SOM portraits of transcriptome profiles across monthly time points from September to July. Each time point is represented by biological triplicates (top rows), with corresponding mean portraits (bottom row). Distinct seasonal expression patterns form two major clusters, “winter-upregulated” cluster (blue box) and “summer-upregulated” cluster (red box). Red areas = upregulated genes; blue areas = downregulated genes. **B** The Group Overexpression Spots SOM landscape reveals cyclic seasonal expression dynamics where each black-edged cluster corresponds to a single spot. Spots are positioned according to dominant overexpression in autumn, winter, spring, or summer, illustrating the annual transcriptional cycle. Labeling is based on individual portraits from panel 2 A. **C** Module correlations (WTO algorithm) show positive (blue edges) and negative (brown edges) correlations between expression modules. The “winter-upregulated” cluster (blue outline) and “summer-upregulated” cluster (red outline) are defined by strongly coordinated modules, with an “intermediate” cluster linking the two main clusters

SOM portrayal provided individual portraits of each transcriptomic sample, where red and blue areas refer to upregulated and downregulated genes, respectively (Fig. 2A). The triplicate samples (for each time point) closely resemble each other throughout the time course reflecting reproducibility, which justified their averaging to obtain mean portraits for each month considered (Fig. 2A). Further averaging over the ‘Winter’ and ‘Summer’ time indicated characteristic clusters of upregulated genes either in the upper right corner or lower left corner of the map. Closer inspection of the individual portraits revealed ten distinct spot-like clusters of upregulated genes (genes listed in Additional file 1) that are summarized in the overview map and labeled with the uppercase letters A-K (Fig. 2B). The self-organizing properties of the clustering algorithm arrange them in a time-dependent fashion, rotating in a clockwise direction throughout the year and thus providing a trajectory of upregulated genes in the gene space. Correlation analysis of the spots revealed mutually correlated winter-upregulated (B-E) and summer-upregulated (G-J) spots (blue lines), which, in turn, were anticorrelated (red lines), thus reflecting antagonistic activation of the respective genes in winter and summer, respectively. Weaker antagonism is found for gene clusters that are upregulated in spring (spot F) and autumn (A and K). Taken together, the sample pattern described in the first section translates into a trajectory of activated gene clusters that are arranged in the SOM portraits in clockwise rotating spot patterns throughout the year.

### Functional annotation reveals seasonally coordinated reprogramming of cellular processes in buds

**Fig. 3:**
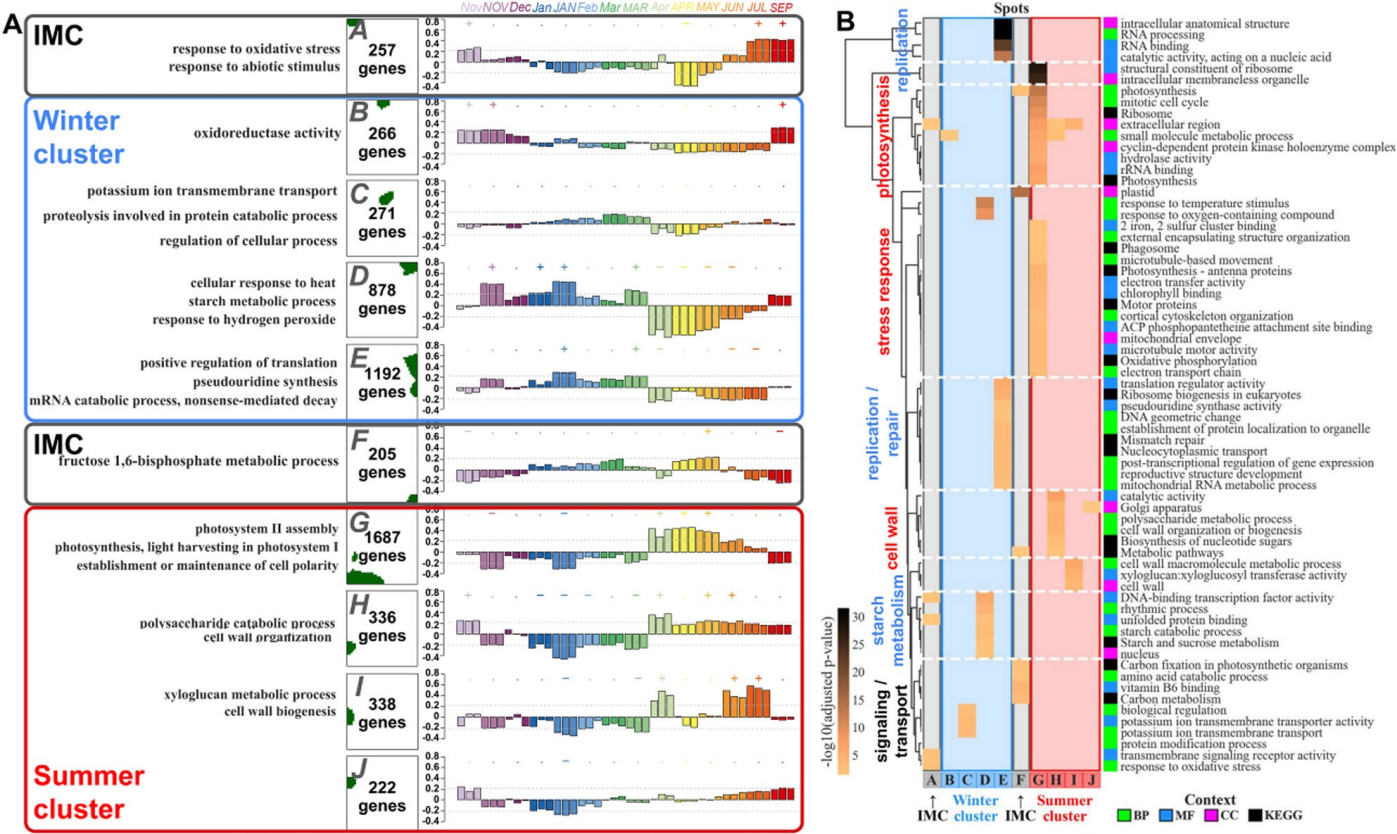
Functional annotation of transcriptomic patterns across bud dormancy and vegetative stages. **A** Ten gene clusters reflecting dominant biological processes based on gene set overrepresentation analysis (left) captured by SOM spots A-J (no enrichment in spot K) with individual SOM portraits (center) indicating the contribution of each gene to the spot pattern and bar plots (right) indicating the relative assignment of each sample to the corresponding spot across the seasonal timeline. Legend: the Y-axis shows z-score normalized relative expression; symbols above bars indicate either significant upregulation (+), downregulation (-) or no significance (·) for each sample. **B** Individual SOM spots (as columns) with enriched functional categories grouped according to biological context, including biological processes (BP), molecular functions (MF), cellular components (CC), and KEGG pathways (as rows), indicated by color coding in the right legend. The color intensity in each cell denotes the significance level of enrichment, expressed as the -log10 adjusted p value, with darker shades indicating stronger enrichment.

Each of the spot clusters contained between approximately 200 and nearly 2,000 grapevine genes and presented a characteristic expression profile across the year (Fig. 3A). Gene set enrichment analysis associated these genes with different functions and highlighted the seasonal reprogramming of grapevine bud transcriptional landscapes between the stress response and energy conservation during dormancy in winter and spring and active metabolic processes during growth resumption in summer. Spots B-E were grouped into the “winter-upregulated” cluster (blue frame), “summer-upregulated” cluster (red frame, spots G–J), and “IMC” cluster (gray frame, spots A, F and K) according to the upregulation of the respective genes in the corresponding months. The “winter-upregulated” cluster is characterized by terms related to oxidoreductase activity, stress responses, translation regulation, and starch metabolism, whereas the “summer-upregulated” cluster is enriched for photosynthesis, cell wall organization, and polysaccharide metabolism, illustrating seasonal transitions in cellular priorities between dormancy and active growth.

We also summarized the significantly enriched gene ontology terms and KEGG pathways among the genes associated with each spot cluster (Fig. 3B), deepening the analysis of the functional landscape of the transcriptomic patterns across the samples. Hierarchical clustering of the SOM spots revealed distinct winter-upregulated (clusters B–E) and summer-upregulated (clusters G–J) clusters, corresponding to the seasonally regulated gene expression profiles. The “winter-upregulated” cluster was characterized by a significant enrichment of GO terms related to replication/repair, proteolysis, and stress response, which are mostly consistent with biological processes typically activated during cold acclimation [25]. In contrast, the summer-upregulated cluster showed enrichment of terms linked to photosynthesis, plastid organization, chlorophyll binding, starch metabolism, cell wall organization, signaling, and transport processes, reflecting active growth and development. Hence, this functional annotation of spot-associated genes provides an overview of the transcriptional programs with emphasis on seasonal adaptation and metabolic reconfiguration in grapevine buds, highlighting the dynamic interplay between replication, the stress response, photosynthetic activity, and metabolic processes across dormancy and active growth periods.

### Thiamine and stilbenoid biosynthetic pathways are triggered during winter dormancy

**Fig. 4:**
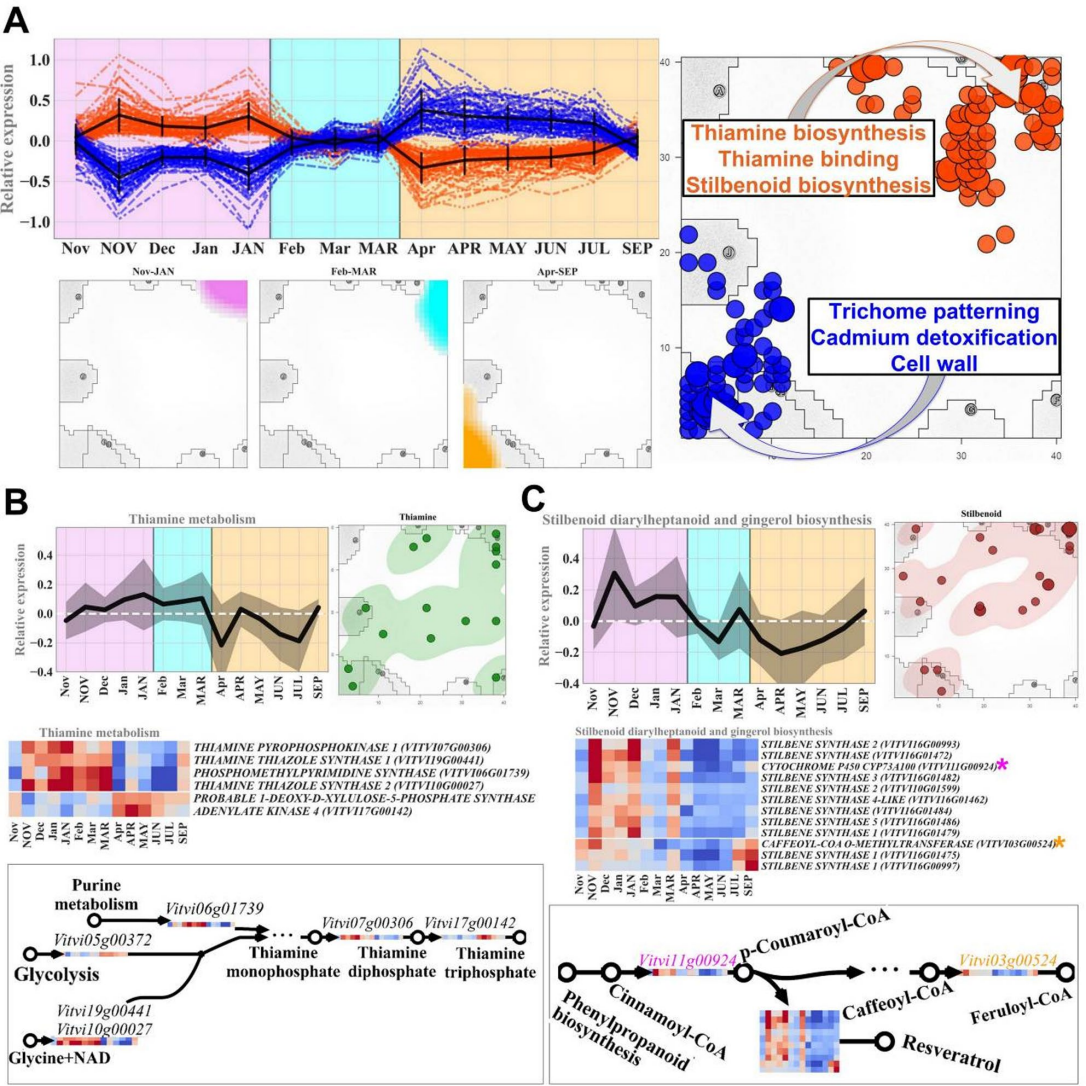
Seasonal regulation of thiamine and stilbenoid biosynthesis during grapevine bud dormancy. **A** SOM profiling of two contrasting expression clusters: the “winter-upregulated” cluster (orange), active from November to April, coincides with bud endodormancy/ecodormancy, and the “summer-upregulated” cluster (blue) aligns with paradormancy and active growth (April–July). Genes from these clusters localize to opposite corners of the SOM, indicating distinct functional programs. **B** Expression dynamics and spatial mapping of thiamine biosynthesis-related genes showing marked upregulation during the winter months, with increased transcription in the middle-winter months, suggesting increased demand for thiamine-derived cofactors during dormancy. **C** Transcriptional patterns of stilbenoid biosynthetic genes, including multiple stilbene synthase (STS) isoforms, exhibit concurrent elevation in expression during the same winter window; their SOM localization supports coordinated activation of stilbenoid defense metabolism in dormant buds. Legend: lineplots: black line = average relative gene expression of all the genes in the pathway, grey intervals = standard deviation; heatmaps: red = gene upregulation, blue = gene downregulation. Samples are displayed according to dataset availability, not strict seasonal sequence

The transcriptional activity of genes of the “winter-upregulated” and “summer-upregulated” clusters revealed antagonistic seasonal expression patterns, with expression spots mapping to opposite corners of the SOM (Fig. 4A). GO enrichment analysis of the two seasonal clusters revealed strikingly divergent functional landscapes. The “summer-upregulated” cluster was enriched in biological processes linked to active tissue development, including “trichome patterning”, “cell wall organization”, and “cadmium ion detoxification”. In sharp contrast, the “winter-upregulated” cluster was strongly enriched for stress-response processes and specialized secondary metabolism. Among the most overrepresented “winter”-specific GO terms were “thiamine diphosphate biosynthetic process” (GO:0009229) and “thiamine binding” (GO:0030975). Importantly, genes of “Stilbenoid diarylheptanoid and gingerol biosynthesis” KEGG pathway were previously shown to be significantly enriched during the winter months ([10, 26], Fig. 3). The “winter-upregulated” cluster pattern suggests that buds in the dormant state channel metabolic activity toward cofactors and secondary metabolites that bolster their capacity to endure cold stress.

## Discussion

In grapevine, axillary buds enter dormancy in response to environmental cues such as shortening photoperiod and decreasing temperatures. Unlike species with terminal buds dormancy, the shoot apical meristem in grapevine continues elongating until conditions become unfavorable [32, 33]. In the Northern Hemisphere, grapevine buds typically transition from paradormancy to endodormancy between late summer and early autumn [6, 34]. Endodormancy becomes fully established by October–November and is generally released only after sufficient winter chilling, most often in early spring.

Our integrated transcriptomic and functional analysis revealed two major clusters. The “winter-upregulated” cluster, spanning November to March, overlaps with the physiological period of endodormancy and ecodormancy in grapevine buds [4]. Buds during this phase are morphologically formed but developmentally arrested, maintained through both internal regulatory programs and environmental cues such as low temperatures and short photoperiod. In contrast, the “summer-upregulated” cluster, extending from April to July, is associated with paradormancy, when apical dominance and active shoot growth suppress lateral bud outgrowth following release from ecodormancy. By reconstructing annual transcriptomic trajectory using SOM-based approach, we capture the continuous seasonal cycle of transcriptional reprogramming, including transitional phases in autumn and spring. This differs from previous studies [10, 11] that analyzed dormancy phases largely in isolation. The SOM projection revealed temporally ordered “spots” rotating through the seasons, providing a dynamic visualization of gene expression and antagonistic regulation between summer and winter programs. This system-level view highlights dormancy as an emergent property of multiple, seasonally coordinated gene modules rather than the effect of a single switch-like mechanism.

A major outcome of our analysis is the consistent and strong induction of thiamine biosynthesis during the dormant months (Fig. 4B). Thiamine (vitamin B1) and its active cofactor TDP support enzymes in central carbon metabolism, including transketolases in the oxidative pentose phosphate pathway, pyruvate dehydrogenase, and α-ketoglutarate dehydrogenase in the TCA cycle, which are essential for energy balance, redox homeostasis, and metabolic flexibility under low-temperature stress [28]. Amplification of thiamine biosynthetic genes increases the cellular TDP pool and may act as a signal enhancing abiotic stress tolerance [16, 35]. In the “winter-upregulated” cluster, we identified upregulation of *Vitvi07g00306*, which encodes THIAMINE PYROPHOSPHOKINASE, the enzyme responsible for converting thiamine to TDP, the biologically active cofactor form [27, 28]. Expression of this gene peaked between December and February and declined sharply as temperatures increased. The observed winter-specific upregulation of the TPK gene and other thiamine-related genes aligns with our initial hypothesis that dormant buds maintain a metabolically conservative yet defense-oriented state. A similar seasonal trend was observed for THIAMINE THIAZOLE SYNTHASE 1 (*Vitvi19g00441*, *Vitvi10g00027*) and PHOSPHOMETHYLPYRIMIDINE SYNTHASE (*Vitvi06g01739*), two key enzymes of thiamine biosynthetic pathway [28]. In contrast, THIAMINE-PYROPHOSPHATE-SYNTHASE (*Vitvi17g00142*), which contributes to the formation of thiamine [28], was not active during mid-winter but instead showed elevated expression during the transition toward active growth. We hypothesize that such a seasonal regulation likely reflects an adaptive strategy in which the thiamine pool (particularly TDP) is increased during dormancy to maintain adequate cofactor availability at the time when low temperature restrict enzyme kinetics [15]. Furthermore, thiamine and its phosphorylated derivatives have been implicated in plant stress signaling pathways, and exogenous thiamine treatment has been shown to enhance tolerance to oxidative stress and pathogen challenge in several species [15, 36]. This mechanism may be directly relevant to the overwintering survival of grapevine buds, which experience sustained oxidative pressure during low-temperature stress.

Interestingly, the expression pattern of thiamine biosynthetic genes indicates an integrated role for thiamine in sustaining carbon fluxes and cofactor availability when enzymatic rates are constrained by cold. Concurrently, stilbene synthase and associated stilbenoid biosynthetic genes show coordinated upregulation during dormancy, providing antimicrobial and antioxidant protection [26, 29, 37]. Our reanalysis also reinforces and extends previous work linking stilbenoid biosynthesis to bud dormancy and cold adaptation [10]. Stilbenoids, particularly resveratrol and its derivatives, are phytoalexins produced predominantly by the *Vitaceae* family. They are known for their potent antimicrobial and antioxidant activities. Previous reports show that resveratrol accumulates in response to pathogen challenge, UV exposure, and oxidative stress [30, 38]. Dormant grapevine buds exhibit reduced metabolic activity and cell division, conditions that generally limit pathogen proliferation; however, episodic stresses such as freezing injury or tissue dehydration can transiently increase susceptibility, making sustained stilbenoid accumulation a relevant protective strategy during winter. The synthesis of stilbenoids is conducted via the phenylpropanoid pathway, where *stilbene synthase* (STS) catalyzes the cyclization of *p-coumaroyl-CoA* and *malonyl-CoA* into the stilbene backbone. In our dataset, the STS-encoding genes clustered within the winter SOM module (Fig. 4C), showing synchronized transcriptional activation from late autumn, with the highest expression occurring in mid-winter. By April, expression levels decreased rapidly, paralleling the shift in metabolic resources toward growth and reproduction [29–31]. Interestingly, several STS isoforms displayed subtly distinct temporal profiles, hinting at possible side functions or different regulations by specific environmental signals, such as chilling requirement fulfillment or photoperiod shifts. These differences could reflect a fine-tuned defense strategy in which certain stilbenoid structures dominate early in dormancy, whereas others are favored during the late stages before bud break.

The results extend prior observations by showing simultaneous activation of thiamine and stilbenoid pathways, rather than examining each independently. This suggests a functional interplay between cofactor provisioning and specialized metabolite defense, likely enhancing redox stability and secondary metabolite biosynthesis under winter dormancy conditions. The simultaneous upregulation of thiamine and stilbenoid biosynthesis during dormancy is broadly consistent with our hypothesis that dormant buds maintain a metabolically conservative yet defense-oriented state, although the degree of coordination we observed was stronger than initially anticipated. TDP-dependent enzymes sustain the carbon skeleton supply and energy balance necessary for secondary metabolite synthesis under metabolically restrictive winter conditions [28, 35]. Through transketolase activity, thiamine supports the oxidative pentose phosphate pathway, the principal source of cytosolic NADPH required for reductive biosynthesis and detoxification of oxidative stress [35, 39]. In parallel, stilbenoids such as resveratrol act as potent antimicrobial and antioxidant metabolites [30, 38, 40]. Thus, the co-induction of thiamine and stilbenoid pathways likely reflects an integrated metabolic reinforcement strategy that enhances both redox stability and biochemical defense during winter dormancy. This may represent a coordinated metabolic strategy rather than two parallel, unrelated responses. These findings align with previous reports showing that thiamine metabolism is activated under cold and oxidative stress [19], and that stilbene accumulation represents a hallmark defense response in dormant or stressed grapevine tissues [10, 20]. However, earlier studies generally examined these pathways independently. In contrast, our data indicate that their transcriptional activation occurs concurrently, providing new insight into an integrated winter defense program.

Our meta-analysis, which combined RNA-seq data from ‘Rosario Bianco’ buds [11] with microarray profiles from ‘Tempranillo’ buds [10], builds directly on the foundations laid by these two studies. Shangguan et al.. emphasized nodal position-dependent variation in dormant buds, whereas Díaz-Riquelme et al.. described expression programs during bud development [10, 11]. By harmonizing and reanalyzing these datasets, our work provides an integrated seasonal map that bridges their findings and identifies additional regulatory dimensions. Specifically, the coordinated upregulation of thiamine and stilbenoid biosynthesis are winter hallmarks. This integrated perspective provides a new insight: dormancy-associated defense in grapevine buds may rely not only on classical specialized metabolites but also on upstream cofactor provisioning, thereby coupling redox homeostasis with secondary metabolite biosynthesis. We acknowledge limitations of our study, which relies on pre-existing datasets from two cultivars and therefore may not fully capture the diversity of transcriptomic responses across grapevine varieties or environmental conditions. Further experimental validation, including functional assays of key genes in thiamine and stilbenoid pathways, is needed to confirm the inferred regulatory relationships and their impact on dormancy resilience. Additionally, integration with proteomic and metabolomic data could strengthen the mechanistic link between transcriptome patterns and metabolic function.

## Conclusions

Grapevine buds employ a coordinated metabolic strategy during winter dormancy, characterized by the upregulation of thiamine and stilbenoid biosynthesis. This dual strategy integrates cofactor provisioning for essential enzymatic activity with the accumulation of protective metabolites, enhancing redox balance and ensuring bud survival under cold and stress conditions.

These findings provide new mechanistic insights into the molecular basis of bud dormancy and stress resilience, identifying potential targets for improving cold tolerance and adaptation in grapevine breeding and cultivation programs. Future work should investigate the regulatory networks orchestrating this coordination, including transcription factors and hormone signaling pathways that mediate seasonal transcriptional switches [3].

## Supplementary Information

Supplementary Material 1. Additional file 1: SOM spot genes.

## Data Availability

The dataset supporting the conclusions of this article is available in the ArmLifeBank repository, https://armlifebank.am/data_files/16. An interactive analysis platform of the dataset is provided by the “oposSOM” browser [41] under the link, http://gondwanaland.izbi.uni-leipzig.de:5978/?dataset=17.

## Abbreviations

SOM: Self-Organizing Map
IMC: Intermediate Cluster
STS: Stilbene Synthase
RNA-seq: RNA Sequencing
GO: Gene Ontology
KEGG: Kyoto Encyclopedia of Genes and Genomes
